# Estimating the costs for the airport operator and airlines of a drone-related shutdown: an application to Frankfurt international airport

**DOI:** 10.1007/s12198-020-00212-4

**Published:** 2020-07-08

**Authors:** Philippe Wendt, Augusto Voltes-Dorta, Pere Suau-Sanchez

**Affiliations:** 1 Management Science and Business Economics Group, University of Edinburgh Business School, Edinburgh, EH8 9JS UK; 2 Faculty of Economics and Business, Universitat Oberta de Catalunya, Av. Tibidabo, 39-43, 08035 Barcelona, Spain; 3 Centre for Air Transport Management, Cranfield University, Cranfield, MK43 0TR UK

**Keywords:** Drone attack, Airport closure, Cost estimation, Passenger recovery

## Abstract

Commercially-acquired drones threaten airport operations due to limited knowledge of airspace safety regulations or deliberate action by drone operators. This study aims to determine whether the investment cost of a drone-defence system can be justified in relation to the financial cost of a drone-related shutdown. To that end, a case study of Frankfurt Airport is carried out with simulations of different disruptions during a peak-activity period similar to the 2018 Gatwick drone incident. With data on passenger traffic and airline schedules, we developed a passenger recovery algorithm to determine the amount of delays caused by the disruptions and the costs for the airport operator and the airlines. Results show that the investment in a drone-defence system is offset by the costs of a 48-h continued closure or several smaller closures, but since the largest share of costs is borne by the airlines, investments should be shared between both stakeholders.

## Introduction

Despite the recent COVID-19 pandemic having an adverse effect in air travel worldwide (Suau-Sánchez et al. 2020), the expected recovery of air transport demand in the medium term (IATA 2020), paired with adverse shocks in the industry, will again place increasing pressure on airport networks to cope with disturbances (Cardillo et al. 2013). Traditionally, these shocks include industrial actions, terrorist attacks and adverse weather events. For example, Hurricane Sandy in 2012 led to airport closures and the cancellation of approximately 180,000 flights (De Langhe et al. 2013). However, a new threat has recently emerged. Drones became a nuisance for airports worldwide as the number of drone incidences is increasing while effective countermeasures are still in their testing phase (McFarland 2019). Initially created for military purposes, drones found their way into consumer and commercial applications. Studies have shown that a drone colliding with a larger aircraft can potentially cause more damage than a bird strike. Additionally, there is also a business risk involved that could cost airports, airlines and passengers several millions of pounds or euros in revenue from delayed and cancelled flights (Hornigold 2019). One of the most significant incidences caused by drone activity occurred at London Gatwick on December 2018, disrupting flights of more than 140,000 passengers (nearly 59% being EasyJet customers) and about a thousand flights (Rawlinson 2019; Detrick 2019). The economic loss from the 33-h disruption was estimated at approximately €55.8 million, primarily to airlines. EasyJet announced a loss of €16.7 million in revenue and customer welfare costs.

Technologies that provide active countermeasures to deal with drone threats in restricted airspaces come at a cost. The technology deployed during the Gatwick incident include a military-grade laser range finder for tracking the drones’ position and a jammer to interfere with signals sent by the operator to the drone (Anon, 2018). This cutting-edge technology has a price tag of €2.9 million per set. They are called *Drone Domes*, of which the British Army purchased six (Duggan 2019). The owners of Gatwick confirmed (Liptak 2019) that they spent millions on equipment (Rawlinson 2019) and installation for their anti-drone defence system. Due to the high costs of anti-drone systems, airports and airlines may want to balance this price with the total cost associated to extreme airport closures. In relation to this, there is a small number of studies (Yan and Lin 1997; Rupp et al. 2010; Gordon et al. 2007) that focus on estimating the economic implications of airport closures for diverse stakeholders resulting from airport closures. However, these studies mainly employ qualitative approaches and there are no past academic studies that estimate the economic costs of airport closures in a peak activity scenario.

We provide a case study for Frankfurt International Airport (FRA) in order to allow for comparison and a discussion on the main cost drivers. In Germany, the number of drone incidences increased from 14 to 158 between 2015 and 2018. 31 events were logged at FRA alone in 2018 (Anon 2019a). The aim of this paper is to determine whether the investment in a drone defence system at Frankfurt airport can be justified in comparison to the total costs experienced by the airport operator due to a complete shutdown for a period of up to 48 h as well as whether the affected airlines would have an interest in contributing depending on their financial impact.

We build on the concepts and frameworks presented in past papers to develop a quantitative approach to estimate costs. We focus on departing passengers during the disruption period to estimate the local effect experienced by Frankfurt Airport and the airlines operating in the German hub. At the core of our methodology, there is a simulation of delays using an algorithm for passenger recovery, which serves as reference to estimate passenger welfare costs. This simulation requires both passenger and airline schedules data for the airport. The airline schedules data is extracted from the Official Airline Guide (OAG) while the passenger itineraries are sourced from the well-known Market Information Data Transfer (MIDT) dataset. Optimal recovery opportunities for each passenger are computed with recovery aircraft including seat capacity restrictions and also high-speed rail. Welfare costs for airlines consist of meal vouchers and hotel stays. Costs for the airport operator relate to loss of landing and passenger charges.

The remainder of this paper is structured as follows. Chapter 2 provides a literature review on drone technology and the costs associated with airport closures. Furthermore, air transport resilience and vulnerability topics will be investigated in order to place our contribution within the existing literature. Chapter 3 describes the methodology in detail, including all assumptions and limitations of our passenger recovery algorithm. Additional information about the case study of Frankfurt airport and all data sources is provided. Chapter 4 summarises the main research findings by providing a breakdown of the costs under different scenarios, as well as the implications for the airport operator and airlines. Chapter 5 concludes with a summary and some recommendations for future research.

## Background and literature review

### The Gatwick incident

Between the 19th and 21st of December 2018, a 33-h closure at UK’s second busiest airport affected up to 140,000 passengers causing more than 1000 flights to be cancelled or delayed (Rowlatt 2019). The operators of Gatwick airport repeatedly tried to reopen the airport. However, on each occasion a drone reappeared, up to a total of approximately 40 times (Douglas 2018), forcing the runways to be closed as per the airport security protocols. The chaos started with the first drone sighting at 21:03 GMT on Wednesday 19th of December. By 10 am on the next day, the police were unable to find neither a connection to terrorism nor that the disruption was not accidentally caused by a pilot (Douglas 2018). At noon, the airport reported that approximately 110,000 passengers were expected to pass through the airport terminals on Thursday 20th of December, a majority of whom would likely experience cancellations and disruptions. In the late afternoon, the British Army was involved to help with the operation as military-grade drone-defence technology was spotted on top of the airport building. Throughout Friday 21st of December, flight traffic was expected to normalise, and the airport was almost back to normal in the early evening. Another drone sighting caused the airport to be closed for yet another hour, but after 6:30 pm all runways remained available for landings and departures.

The 33-h disruption during the Christmas peak travel period resulted in costs of approximately £50 million (Calder 2019; Detrick 2019). According to Calder (2019), the grounded flights costed EasyJet £5 million (€4.5 million) in lost revenue and almost £10 million (€9 million) on customer welfare costs (meals, accommodation, and alternative transport). Many passengers stranded in the terminals had to sleep on benches or even on the terminal floor. Extending the impact proportionally to other airlines, the total damage can be estimated at between £35 to £40 million (€31.5 to 36.0 million). Initial figures from January 2019 estimated the impact for the airport at around £15 million (€13.5 million) but reports from June 2019 claim the chaos just cost the airport £1.4 million (€1.3 million) (Menendez 2019; Topham 2019) because airlines ultimately took most of the fall for the incident.

Gatwick may not be the last airport drone incident, but it was the most significant in terms of economic damage. Unless counter-drone systems are used more frequently around the world, incidents like this might happen again. The market already provides a handful of counter-drone technologies (Stokel-Walker 2019). However, all these systems are costly. Thus, airports may want to balance the cost of a drone defence system with the costs of an airport shutdown.

### Available drone-defence systems and investment costs

Flying drones near airports endangers the safety of passengers and flight crew onboard an aircraft. The probability that the drone is drawn into the turbine is small, but its battery poses a potential risk of fire inside the turbine as it may not shatter on impact. Furthermore, due to the high relative speed of collision, a drone with two kg of mass could exert enough force to break the windshield of some aircraft types, resulting in the loss of cabin pressure (Porter 2010).

In view of the potential risks, authorities are increasingly interested in the development of technologies to counteract illegal drone activity (counter-drone technology), also known as counter-unmanned aerial vehicles (C-UAV). The market for C-UAV is currently estimated to be worth between 500 million to one billion dollars (Wright 2017). The most common methods used to counter UAVs are frequency jamming, spoofing, laser, nets, counter-drone and projectiles. Frequency jamming technologies disrupt the link between the operator and the drone, forcing the vehicle to descend or return home. Hijacking a drone’s communication system is referred to as “spoofing” and allows the user to take over control of the target drone. A laser beam system can provide enough energy to partially or completely destroy vital segments of a drone to stay airborne, causing it to fall to the ground. Nets can also be employed to entangle a target drone making it unable to remain airborne. A counter-drone system (Dedrone 2019) involves deploying a separate drone to destroy or disrupt a target drone resulting, most commonly, in the loss of both drones. The organizations that adopt this technology commonly use a combination of interception elements.

According to Pinkstone (2018), the equipment employed at Gatwick was a thermal and optical laser rangefinder system able to track drones in a radius from three to six miles. This device is connected to a radio frequency jamming system (Bravo 2019), which was complemented with a six-directional radar able to pin-point the drone operator once inside the perimeter range. Both systems work together and are known as a “Drone Dome”. Initial reports (Duggan 2019; Pinkstone 2018) claimed that the British Army spent £15.8 million (€17.6 million) for six Drone Dome systems, which gives a unit price of £2.6 million (€2.9 million). Early reports from January 2019 state that Gatwick Airport spent £800,000 (€720,800) (Apen-Sadler et al. 2019) while reports from June 2019 claim that the airport ended up spending £4–5 million (€3.6–4.5 million) (Menendez 2019; Topham 2019; BBC 2019) on anti-drone technologies. This would be the reference value for the minimum investment necessary to protect Frankfurt Airport in our case study.

### Costs of airport closures

An airport shutdown is defined as a situation when an airport is unable to offer its full capacity due to exogeneous reasons (De Langhe et al. 2013; Rupp et al. 2010). Shutdowns may be total or partial and can originate from infrastructure problems (weather or technical problems) or human factors (security). According to De Langhe et al. (2013) there are two key differences between weather and security-related shutdowns. First, weather-related closures can, to a certain degree, be predicted in advance such that the airport can take pre-emptive measures while security shutdowns can, almost certainly, not be predicted and leave the airport more exposed. Finally, weather conditions may affect multiple airports that are geographically concentrated in a region while security incidents usually affect only a single airport at a time. After the decision of closing the airport has been made, the operator will immediately send out a Notification to Airmen (NOTAM) to the airlines, as well as informing the passengers as soon as possible. Based on past events involving drone-related airport closures (Taylor 2018; Deutsche Welle 2019), the standard reaction is to close the affected airport initially for 30 min, which, in most cases, exceeds the average flight time of commercially available drones (BBC 2018). After reopening, the airport may operate at reduced capacity because of the missing capacity from aircraft that were not able to land during the closure period. Furthermore, the unpredictability of the drone attack may lead to a total closure rather than a partial one. A second difference with other types of security shutdowns (such as a terrorist attack) is the ability of the airport to reopen at any time after the drone has been neutralized if there is no physical damage to the infrastructure. This can make the management of passenger terminals more difficult as passengers are less likely to leave the terminal (or stop coming in) if flights can resume at any time. On the other hand, being a concentrated event, other nearby airports remain unaffected and available to serve the disrupted passengers.

Identifying the affected stakeholders is a key step in measuring the effects of an airport shutdown. Maertens (2013) mentions that such type of event can affect not only the air transport value chain but also the entire economy of the affected country, particularly if the airport under attack is a primary hub. Within the aviation sector, De Langhe et al. (2013) mention the airport operator, airlines, passengers, and service providers. The focus for this research will be on the first three due to the lack of data on the latter. There is a fair number of studies on the determination of the economic impacts of airport closures (Yan and Lin 1997; Rupp et al. 2003; Gordon et al. 2007; Maertens 2013; Serrano and Kazda 2018; Pejovic et al. 2009). Based on these studies, we decided to implement a bottom-up approach considering some of the airport- and airline-associated costs indicated in Table 1.

**Table 1:** Airport- and airline- related costs during an airport closure

Airport	Airline
Loss of aeronautical revenue	Loss of revenue from ticket sales
Loss of non-aeronautical revenue	Cost of grounded aircraft
Increased labour and material costs	Customer welfare expenses and compensation

Source: Own elaboration

Airports will experience losses in terms of aeronautical revenues, which depend on traffic volumes that become disrupted during the shutdown (Maertens 2013). For example, in Frankfurt Airport, the operator charges a landing fee according to the maximum take-off weight of the relevant aircraft, and another fee per departing passenger depending on the flight destination. Losses in non-aeronautical revenues (e.g. retail, food, etc.) will be experienced if the airport perceives a fixed percentage of revenues from the concession. Maertens (2013) states that labour and material costs will also depend on passenger volumes yet only in the long run, since most airport expenses can be considered fixed in the short run. However, major disruptive events can result in increased terminal facility expenditures.

In regard to airlines, the European Union (EU) legislation 261/2004 (EC 2004) grants passengers the right to claim compensation in the event of flight delay or cancellation. However, air carriers are not liable to pay passenger compensation if the “cancellation occurs in extraordinary circumstances which could not have been avoided even if all reasonable measures had been taken” (EC 2004). In the Gatwick case, the UK Civil Aviation Authority confirmed that the drone incident was an extraordinary circumstance and airlines should not be obliged to pay compensation (Morris 2018). Nevertheless, the airline is still liable to provide their passengers with accommodation (for overnight stays), meals and refreshments, appropriate means of communication, and airport transfers. This is unless the passenger decides to cancel their trip, so their behavioural choices affect airline costs. Following Maertens (2013), passengers affected by a short interruption of a few hours can be expected to continue their trip. When shutdowns increase in duration, the proportion of passengers switching to other modes of transport or cancelling their trip increases. In medium to long-haul flights, cancellations are typically lower due to road or rail alternatives being generally inconvenient. On the other hand, passengers with short-haul flights typically either cancel their trip entirely or switch to other mode of transport to their destination like rail. Business and leisure passengers show differences in the acceptable delay experienced during a travel disruption. On average, business passengers show lower tolerance for flight delays compared to leisure passengers (Jiang and Ren 2019). In the end, the airline can also decide to cancel the flights directly and offer passengers a refund, so welfare costs are limited by airfares.

All of the metrics above depend on an assessment on passenger delays, which, in turn, depend on how vulnerable the affected airport is to the disruptive event and how much time it requires to resume normal operations (i.e. resilience).

### Airport resilience and vulnerability

Vulnerability can be defined as “a susceptibility to incidents that can result in a considerable reduction in network serviceability” (Berdica 2002). This concept can be linked to two elements: the probability of a disruptive event occurring and the damage the event causes (Jenelius et al. 2006). Faturechi and Miller-Hooks (2015) list dozens of published works about the resilience of transport networks. An established approach to determine system damage and vulnerability is to model the redistribution of traffic flows under disruptive events by means of user re-routing algorithms (Jenelius et al. 2006; De los Santos et al. 2012 or Rodríguez-Núñez and García-Palomares 2014). This allows for disruption costs to be calculated from the users’ perspective by comparing their original and altered itineraries. Translating these concepts into an aviation network means to gather information about airline schedules and passenger bookings and then modelling how the carriers adjust to disruptions by delaying and cancelling flights, relocating disrupted passengers, and reassigning crews and aircraft to different routes (Barnhart 2009). As metrics of system damage, we can mention the number of disrupted passengers, the percentage of those passengers relocated, and the difference in time between the original and the rescheduled itineraries which equals the total delay experienced (Bratu and Barnhart 2006).

Studies that develop a passenger relocation algorithm to investigate the vulnerability and resilience of air transport networks are scarce in the literature. Moreover, most studies do not incorporate actual data on passenger bookings. For example, Cardillo et al. (2013) designed a passenger rescheduling algorithm simulating random failures in an air transport network to reassign affected passengers to alternative itineraries and determine the changes in the network’s topological indicators. Hossain et al. (2013) developed a rescheduling algorithm to account for airport capacity and ground transfer times between airports in Australia. The resulting unit cost of relocation from each airport closure was used to rank airports according to their vulnerability. Furthermore, the authors found that having alternative airports to recover stranded passengers helped to mitigate the impact of the closure.

Finally, Voltes-Dorta et al. (2017a) built on past contributions to investigate passenger recovery after an airport closure. The paper outlines a shortest-path-length algorithm that finds the optimal alternative routing for all disrupted passengers while also considering capacity constraints. The study combined both airline schedules and passenger booking data. This is important because the bookings indicate the full passenger itineraries while the schedules allow for recovery options to be established. The impact of each investigated closure scenario is determined by the proportion of passengers for whom an alternative itinerary within the recovery period was found (relocation rate) and the average departure delay experienced by the passengers. However, due to the lack of data, the authors did not attempt to translate these measures into monetary costs. Their method is suitable for our paper since it allows us to estimate airline costs in terms of passenger welfare as several passenger rights are triggered by delay thresholds. The proportion of disrupted passengers who are entitled to care, the number of overnight stays and the proportion of passengers that would require ground transfers can be calculated.

By ranking all major European airports using the method above, Voltes-Dorta et al. (2017a) concluded that Frankfurt stands out from the rest in terms of operational resilience, having the highest relocation rate of 86.9% and the shortest average delays after a hypothetical 24-h closure of the main German hub. This was linked to three factors: 1) the dual-hub operation of Lufthansa at Frankfurt and Munich that can provide hub alternatives for connecting markets. 2) Frankfurt’s good connectivity to road networks offering short transfer times to nearby airports. 3) A high share of alliance traffic, which enables Lufthansa to find alternative travel plans for its disrupted passengers in flights of alliance partners. A faster passenger recovery translates into lower damage costs, which makes the choice of Frankfurt a suitable one to deliver a lower-bound estimate for the costs of a drone attack at a major hub airport.

Still, the recovery algorithms developed by past authors lack the inclusion of rail alternatives for passengers leaving to nearby airports or cities which, if included, would make for a more realistic approach. Additionally, the authors assumed that all passengers decided to travel (i.e. no tickets got cancelled) despite the long waiting times. Also, all flights retained their original schedules so flight delays were not modelled. Finally, they did not consider that airlines may decide to increase aircraft capacity after the airport reopens to speed up passenger recovery.

### Contribution

To the best of the authors’ knowledge, this will be the first study that investigates the financial consequences of a drone-related airport closure for the airport operator and airlines by performing a simulation of passenger recovery using actual data. From a methodological perspective, we add to existing literature on airport resilience and vulnerability because we improve on existing passenger recovery algorithms to obtain a more precise estimate for the airline and airport costs. The improvements are the inclusion of rail recovery options for affected passengers, as well as the implementation of different behavioural assumptions for passengers.

## Data and methodology

The main goals of the methodological process are (1) to determine the number for passengers waiting in the terminal facilities of the airport during the simulated closure, (2) compute optimal recovery opportunities for each passenger and (3) to calculate total costs resulting from the simulated closure, with focus on customer welfare and loss of revenue for the airport.

### Case study

Frankfurt airport (FRA) is Germany’s largest airport and it is owned by Fraport AG. It is Europe’s third-largest hub airport and it has its own long-distance rail station (in the airport’s lower floors) that connects the airport with Europe’s high-speed rail network operated by the Deutsche Bahn. The dominant airline is Lufthansa, the German flag carrier. Due to its location in central Germany and good accessibility, it is relatively close to other major airports such as Duesseldorf, Stuttgart, or Munich. Consequentially, there are many airports that could function as an alternate airport in the event of a complete shutdown at FRA. The annual passenger traffic at the airport increased to 69.5 million in 2018 (Fraport 2018a), representing a growth of almost 8%. Our method focuses on departing passengers from Frankfurt (as in Voltes-Dorta et al. 2017b) in order to calculate the “local” costs of the disruption. The top three domestic destinations are Berlin, Hamburg, and Munich, while the top European destinations are London, Vienna, and Madrid. Top destinations in Asia include Dubai, Shanghai, and Seoul while New York, Toronto, and Chicago are the top destinations in North America. At Frankfurt, the share of business travellers in 2017 was 36% while leisure accounted for 64% (Fraport 2018b).

### Datasets

The method combines supply data (airline schedules and seat capacities) and demand (passenger bookings and travel itineraries). The supply dataset is sourced from OAG Schedules and includes all flights that take-off and land in European airports between the 18th and the 28th of December 2017. This period was selected because it contains the dates of the London Gatwick closures (albeit a year earlier) and it is the period before Christmas when many people travel for holidays and/or return to their families. The demand side is covered by a Marketing Information Data Transfer (MIDT) dataset (sourced from OAG as well) that provides information about passenger bookings and travel itineraries passing through FRA during the focus week. The MIDT dataset consists of 675,095 records of 1,115,203 passengers travelling to 250 direct destinations severed from FRA. Each record contains information about the markets served, passenger booking class, passenger nationality (proxied by the country of sale of the ticket), flight routing, operating airline and flight number, airline alliance, origin and destination airport code, flight distance, number of seats, and departure and arrival times.

Other datasets were collected: 1) average airline ticket price from all three booking classes (first, business, and economy) for short-, medium-, and long-haul flights from FRA. This information was obtained from OAG; 2) maximum take-off weight (MTOW) information for all aircraft types that departed in the focus week to calculate relevant airport charges. This was also sourced from OAG; 3) airport coordinates to determine flight distances from FRA to all served airports obtained from Google Maps; 4) reference values for welfare expenses per passenger (including hotel, vouchers, and meals) were taken from the EasyJet website; 5) the weighted average passenger group size (relevant to calculate hotel expenses) was taken from a 2017 passenger survey referring to Heathrow airport conducted by the Civil Aviation Authority (CAA 2017); 6) the schedule of train departures from the FRA long-distance rail station towards major German cities was obtained from Deutsche Bahn; 7) the official 2019 Fraport airport charges were sourced from the operator’s website (Fraport 2019); and 8) the distance and travel time by road to alternative airports was collected from Google Maps.

### Closure scenarios

**Fig. 1:**
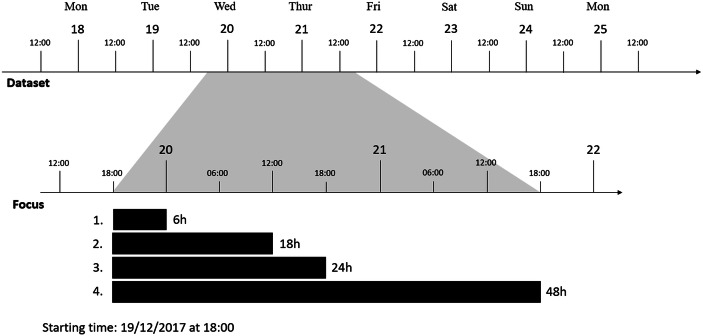
Overview of the four closure scenarios. Source: Own elaboration

We selected four closure scenarios after a hypothetical drone sighting at FRA, all starting at 18:00 UTC + 1 on the 19th of December 2017, with a duration of 6, 18, 24, and 48 h for scenarios 1, 2, 3, and 4, respectively, see Fig. 1. The starting time of 18:00 UTC + 1 was selected because a six-hour closure will cause passengers to stay overnight as the airport closes anyway at 22:00 UTC + 1 due to noise restrictions. As a result, airlines will be facing hotel and meal welfare expenses even in the shortest scenario.

### Passenger behaviour

**Fig. 2:**
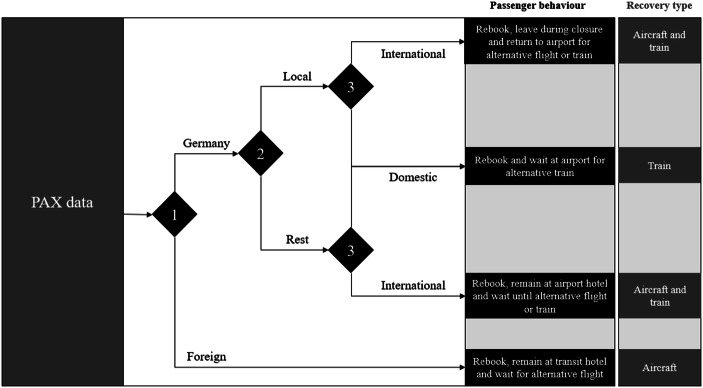
Assumed passenger behaviour during the closure. Source: Own elaboration

Because not all necessary data was available, several assumptions are needed. All passengers will arrive to the terminal two hours before their scheduled or relocated departure times, even during the closure period. This is to reflect the passengers’ perception that the airport can reopen at any time after the threat is neutralized. We know there are different categories of passengers according to the place of residence (“local” Frankfurt travellers, non-local “rest of Germany”, and foreign) and flight destination (domestic or international). It was assumed that 50% of all German passengers travelling internationally from FRA are local residents from Frankfurt am Main or neighbouring towns. The behavioural assumptions are summarized in Fig. 2. All German passengers (local and non-local) can be offered either rail or air transport itineraries (train being the preferential recovery mode for domestic travel), while foreign passengers will only have air transport available. German passengers travelling internationally may be offered a rail transfer to another German airport to continue their travel from there. Local passengers travelling internationally are assumed to leave the airport terminal after a 3-h delay and return to the terminal two hours before their new departure time. All other passenger categories, including non-locals and foreign travellers, remain in (or around) the terminal until their new departure time. Finally, we assume that there are only leisure travellers during the sample Christmas period. This includes all business travellers who are likely to be returning home. This implies that all passengers are expected to accept their alternative flight itineraries, regardless of delay, due to their higher flexibility in terms of departure times in comparison with business travellers (Alderighi et al. 2016).

### Recovery assumptions

**Fig. 3:**
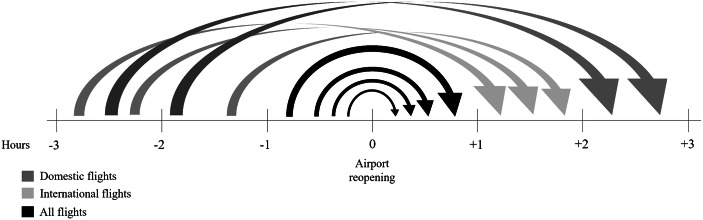
Slot allocation for delayed departures. Source: Own Elaboration

We assume that the airport cancels all flights until three hours before reopening, at which time flights can be delayed. Figure 3 provides an outline of the slot allocation logic for the delayed departures. Flights scheduled to depart in the hour before reopening will be moved to the first hourly slot. International and domestic flights departing between one and three hours before reopening will be allocated to the second and third hourly slots after reopening, respectively. Passengers can only be offered alternative itineraries if seat capacity is available. After reopening, full-service airlines are assumed to increase seat capacity in a fixed percentage (e.g. 10%) by deploying larger aircraft models to speed up the recovery process.^1^

Finally, we define three options available for airlines to re-allocate their passengers. The “Airline” option means that only their own flights can be used. “Alliance” means that affected carriers can tap into the seat capacity of their alliance partners (Oneword, Skyteam, or Star Alliance). Lastly, “All” means that passengers will be offered the next available flight to their destination from any airline with free capacity. The Alliance option is be the default one.

Some passengers can also be offered a direct high-speed rail connection to 19 cities reachable without changes from the airport’s long-distance rail station. Cities in Germany include Munich, Berlin, Hamburg while European cities selected include Amsterdam, Basel, and Brussels. Passengers can also take onward flights from any other German airport reachable by train from Frankfurt. No seat capacity restrictions are set for high-speed rail travel segments due to the large number of departures available from the airport station.

### Relocation algorithm

The algorithm was written in Virtual Basic for Application (VBA) language and executed as an Excel macro. It is based on three data frames: PAX includes the original passenger records, the ALTERNATIVE FLIGHTS data frame includes all available recovery itineraries, and SEAT CAPACITY indicates the available seat capacity in each itinerary. After setting the parameters of the closure, the records in PAX are labelled as “affected departure”, “affected arrival”, or “unaffected”, depending on whether the flight was scheduled to depart or arrive during the simulated closure. The “affected departure” records are then sorted by departing time so that seat capacity is allocated first-come-first-served. The SEAT CAPACITY data frame is built by aggregating the original PAX records to determine the free seats available per flight over the sample week. The ALTERNATIVE FLIGHTS data frame contains all unaffected feasible flight itineraries (depending on the recovery option, e.g. “Alliance”) as well as the rail itineraries out of FRA and the selected alternate airports in Germany.

Table 2 summarizes the steps of the passenger recovery algorithm. First, all German passengers travelling internationally will be randomly assigned, depending on the probability of them being local (e.g. 50%), to leave the airport during the closure and return to their homes. The search for a new itinerary is structured as a loop that runs through each affected PAX record. For all German passengers travelling domestically, a direct train connection will be searched first in ALTERNATIVE FLIGHTS, see step 3a. Should no train be found, and for all other passenger categories, the macro will directly search for the next possible flight connection to the destination airport indicated in the relevant PAX record (3b). Another match function, step 3c, will now check this exact flight in the SEAT CAPACITY data frame. Should there be a free seat available, the capacity will be reduced by one and the found alternative flight will be added to the PAX record (indicating flight number, operator, and new departure time) closing the loop. If, however, there was no free seat available, the algorithm will repeat steps 3b and 3c, explicitly discarding the last solution, as many times as necessary until a seat is found or no alternative flight could be found within one week after the airport reopening, in which case the PAX record will be updated indicating that neither flight nor train options could be found. Steps 2a to 2c and 3a to 3c in Table 2 will repeat in the same manner for all four closure scenarios.

**Table 2:** Passenger recovery algorithm

Steps	Location	Actions
1a	Passenger recovery \ Cale PAX recovery	Clean range containing previous alternative flight searches.
1b	Passenger recovery \ Cale PAX recovery	Set all German passengers traveling domestically to “leave”.
1c	Passenger recovery \ Cale PAX recovery	Randomly assign “leave” to German passengers traveling internationally in 70%, 50%, or 30% of the cases based on the selection made, all remaining will be assigned “stay”.
Start loop 1		
2a	Passenger recovery \ All	Set current scenario (1–4) in the dashboard, refresh all worksheets, and filter ranges in descending order in alternative flights
Start loop 2		
3a	Passenger recovery \ Cale PAX recovery and Passenger recovery \ alternative flights	For passenger X, search (match function) for the next possible available flight (based on origin, destination, operating airline, and departure date) available without considering seat capacity restriction nor the passengers’ origin but only on the first run cycle. Destination airport must however remain the same.
3b	Passenger recovery \ Cale PAX recovery and Passenger recovery \ alternative flights	For the same passenger X, search (match function) for the next possible available flight with the restriction of the same airline or alliance or none of the listed restrictions based on the selected recovery option at the beginning. Searching for origin, destination, operating airline/alliance/none, and departure date. Destination airport must however remain the same.
3c	Passenger recovery \ alternative flights and Passenger recovery \ seat capacity	Check (match function) the seat capacity from the previously found flight. If seats are available, continue next and write the record found in the corresponding passenger record in the “calculated PAX recovery” worksheet. If not, go back to the beginning (3b) and search for another flight five minutes later.
End loop 2		
2b	Passenger recovery \ alternative flights and Passenger recovery \ Cale PAX recovery	If the passenger is German and **no** seat is found, search (match function) for the possible available train keeping the destination airport the same. If a train is found, write the record in the corresponding passenger record in the “calculated PAX recovery” worksheet. If no train was found, write “no available flight nor train found” in the corresponding passenger record in the “calculated PAX recovery” worksheet.
2c	Passenger recovery \ alternative flights and Passenger recovery \ Cale PAX recovery	If the passenger is not German, write “no flight alternative found overall” in the corresponding passenger record in the “calculated PAX recovery” worksheet.
End loop 1		

Source: Own Elaboration

### Determination of costs

Lost revenue for Frankfurt airport is based on “affected arrivals” and their associated passenger-related charges. Departing passenger charges for non-recovered trips are lost as well. Landing charges are €1.90 per ton of aircraft Maximum Take-off Weight (MTOW); passenger charges are €18.16 per departing passenger for domestic and EU flights, €22.84 for non-EU flights, €25.16€ for intercontinental flights, and €12.93 for transfer passenger to all destinations. Security charges are 1,24€ per departing passenger (Fraport 2019).

The recovery algorithm, in combination with the behavioural assumptions, determines the time each affected passenger will stay within the airport terminals before their alternative flight. For non-local passengers with no alternative flight, a maximum stay of five days was assumed. Airline costs predominately arise from customer welfare expenses which consist of vouchers, meals, and accommodation. Using EasyJet compensation guidelines as an approximation, vouchers worth €4.5 are distributed to passengers on flights up to 1500 km every two hours and for flights over 1500 km every three hours. Meals at hotels include an evening dinner and breakfast for €25 per person per day (EasyJet 2017). Hotel night stays are estimated at €90 per room per night based on average online pricing (Anon 2019b). Passengers travel in a weighted average group size of 1.657 people, based on the CAA passenger survey from London Heathrow (CAA 2017). Thus, hotel costs per person are reduced to €54.3 per person per night. It was also assumed that nearby hotels have enough room capacity. If welfare expenses exceed the average ticket price for a given passenger, then the airline decides to refund the ticket instead. According to OAG data, the average return ticket for long-haul flights during that period was €1013.80, for medium-haul it was €318.66 and for short-haul flights it was €269.21. Thus, passenger welfare costs are effectively capped by these amounts.

Besides the airline and airport costs, we also calculate the average delay experienced by the passengers, and the percentage of disrupted passengers non-relocated to alternative flights.

### Sensitivity analysis

We carry out a sensitivity analysis to explore the impact on the estimated airport and airline costs of variations in four key simulation parameters: a) the “waiting hours” denotes the time local passengers stay in the terminal before leaving to wait for their new itinerary elsewhere, b) the % of local German passengers, c) the “capacity response” parameter indicates the percentage increase in seat capacity deployed by airlines to speed up recovery after the airport reopens, and d) “recovery” indicates the options available for airlines to recover the passengers (“Airline”, “Alliance”, or “All”). A total of seven cases (labelled from A to G) were created by modifying the parameters (Table 3). Case D is the central case for the purposes of this research, and it assumes 3-h waiting time, 50% local Germans, 10% extra capacity, and Alliance recovery as described in the sections above. Reference to the other cases will be made concerning the total cost of passenger welfare expenses ranging from the worst-case A to the best-case G. The combination of 7 cases and 4 closure scenarios means that 28 simulations will be run.

**Table 3:** Different cases for sensitivity analysis

Worst-Best	Cases	Waiting hours	% of local Germans	Capacity response	Recovery
Worst	A	4	30%	0%	Airline
	B	3	30%	0%	Airline
	C	3	30%	10%	Airline
Central	D	3	50%	10%	Alliance
	E	2	50%	10%	Alliance
	F	2	50%	20%	Alliance
Best	G	2	70%	20%	All

Source: Own Elaboration

### Limitations

In real-world circumstances, many passengers would be offered military beds (that airports have for emergencies), which can reduce costs for the airline compared to those assumed in this simulation. Perhaps the most significant limitation is that passenger behaviour is nearly impossible to predict as it can depend on their stress tolerance, age, group size, or travel purpose. Furthermore, the proposed method does not consider aircraft or crew positioning or working hour restrictions. Finally, we do not account for group recovery due to data limitations but only find alternative itineraries for individual travellers.

## Results and discussion

An overview of the estimated loss of revenue for the airport operator across the “central” cases (D) of each of the four closure scenarios is shown in Table 4. This includes the loss of departure and landing charges, passenger handling charges, and passenger security charges. The results for the other cases are shown in Appendix Fig. 5, but the variability across cases does not affect the main conclusions of the study.

**Table 4:** Overview of the lost revenue for the airport across all four closure scenarios (central cases)

Loss revenue factors (€)	Scenarios			
	1 (6 h)	2 (18 h)	3 (24 h)	4 (48 h)
Departure / landing charges	74,066	197,154	288,547	574,834
PAX charges	252,885	870,292	1,283,677	2,524,811
PAX security charges	20,801	59,297	98,017	197,562
Total loss of revenue	**347,752**	**1,126,743**	**1,670,241**	**3,297,206**
Loss of revenue per hour	57,959	62,597	69,593	68,692

The loss associated to Scenario 1 (six hours) was approximately €0.35 million. For Scenarios 2 and 3 the resulting lost revenue for FRA was approximately €1.13 million and €1.67 million, respectively. For the most extended simulated closure, the lost revenue increased to €3.3 million when compared to Scenario 1. Referring to Table 5, by far the most significant loss for the airport are passenger handling charges making up on average 76% of the total lost revenue. Departure and landing charges made up an average of 18% and passenger security charges accounted for an average of 6% of the total revenue loss.

**Table 5:** Breakdown of airport losses across all four closure scenarios (central cases)

Loss revenue factors shares	Scenarios				Average
	1 (6 h)	2 (18 h)	3 (24 h)	4 (48 h)	
Departure / landing charges	21%	17%	17%	17%	18%
PAX charges	73%	77%	77%	77%	76%
PAX security charges	6%	5%	6%	6%	6%

Considering that our methodology leads to lower-bound cost estimates, the cost for installing a counter-drone system would already be justified. Still, a single system worth €2.9 million would not cover the entire perimeter of the airport. A two-system investment of €5.8 million would not be justified by a single 48 h event, but, considering the frequency of past drone sightings at FRA, it may end up delivering good value over time. With an approximate loss of revenue of €1.5 million per day, the suggested two systems for Frankfurt would be justified after 89.6 h or 3.8 days of closure.

**Fig. 4:**
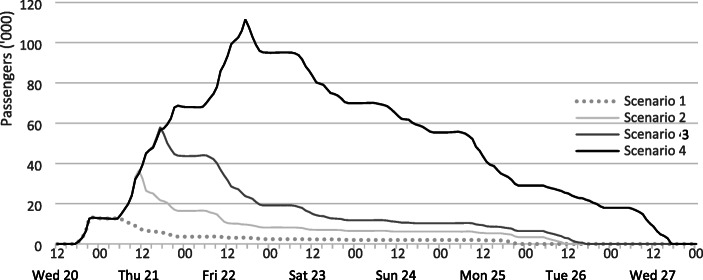
Hourly evolution of disrupted passengers in the terminals during the recovery window (central cases)

Total passenger welfare costs across the “central” cases (D) of each of the four closure scenarios are shown in Table 6. The cost for Scenario 1 is approximately €2.3 million, while the longest closure generated expenses of almost €34.30 million. This value comes close to the estimates from the Gatwick incident of approximately €38–44 million (Calder 2019). Clearly, the most important cost factor are hotel stays, which account for more than half of the costs in all scenarios. Also, airline costs are not linear and costs per hour increase between the first and second day of closure. In the 24 h closure, the average cost per hour is €0.46 million, and in the 48 h closure, the average cost increases to €0.71 million per hour. This is due to the accumulation of passengers as we assume that they keep arriving to the terminal, even during the closure period, due to the uncertainty about reopening and the rate of recovery (by road or rail) being very low in the first hours (Fig. 4). Overall, it takes between six to seven days for the airlines to provide an alternative travel plan to all stranded passengers. Almost 111,000 passengers accumulate in the terminals on the second evening of the simulated closure in Scenario 4, just before the airport reopens for regular operation. Thus, airlines clearly want to provide passengers with alternative flights or a refund as quickly as possible to reduce their welfare expenses.

**Table 6:** Overview of the airline costs across all four closure scenarios (central cases)

Loss revenue factors (€)	Scenarios			
	1 (6 h)	2 (18 h)	3 (24 h)	4 (48 h)
Passenger welfare costs	2,288,378	6,264,033	11,084,449	34,279,860
Costs per hour	381,396	348,001	461,852	714,163
Hotel	55.30%	55.10%	53.40%	52.90%
Meals	25.50%	25.40%	24.60%	24.30%
Voucher	19.20%	19.60%	22.00%	22.80%

Unsurprisingly, Lufthansa is the most affected airline. Looking at Scenario 4, Lufthansa alone transported 81,257 passengers compared to 6559 with Ryanair and 4660 with Condor. Therefore, Lufthansa accounts, on average, for approximately 54.1% of all welfare expenses in each simulated scenario as seen in Table 7. This suggests that Lufthansa could have a high interest in contributing to the acquisition of a counter-drone system to reduce the cost experienced in case of future events.

**Table 7:** Overview of the Lufthansa costs across all four closure scenarios (central cases)

Scenario	Lufthansa welfare expenses (€)	Percentage of total
1	1,300,299	56.8%
2	2,954,679	47.2%
3	5,279,962	47.6%
4	18,555,856	54.1%

When investigating the role of intermodality in the recovery process, we found that only 4.5% of passengers took a rail option. One may argue this is rather low, given the unlimited capacity we assumed, but this could be explained because German passengers represent around 30% of all passengers and, of those, 85% travel internationally during the sample dates.

Table 8 shows the average delays per passenger and non-relocation rates across all four closure scenarios. 9.5% of all passengers were not recovered in Scenario 1 increasing to 13.9%, 14.7%, and finally to 28.3% for Scenarios 2, 3, and 4, respectively. The results for all other cases are shown in Appendix Fig. 6. When evaluating all cases, it seems that the increase in seat capacity had the most significant effect on the reduction of cost as seen from the difference in cases B to C and E to F. It should be noted, however, that this recovery option only applies to full-service carriers because those are assumed to have larger aircraft in their fleet that could be used during the recovery process. As Lufthansa is the primary operating airline at Frankfurt, both alliance support and increasing seat capacity will have an important impact in reducing the costs. The average departure delay experienced by the passengers at FRA in the worst-case closure scenario of 48 h was 74.8 h while for 24 h it reduced to 45.3 h, which explains the large welfare costs for the airlines (Table 8).

**Table 8:** Average delays per passenger and non-relocation rates across all four closure scenarios (central cases)

Simulated Scenario	1	2	3	4
Average delay experienced per passengers (h)	36.3	37.9	45.3	74.8
Non-relocated passengers (%)	9.5%	13.9%	14.7%	28.3%

Looking at the origin airport of the affected arriving passengers and the destination airports of affected departing passengers, we find that the top-five affected airports for the longest closure of 48 h are Heathrow, Madrid, Vienna, Bangkok, and Dubai in descending order. Those airports might experience minor passenger accumulations in the terminals as departing flights to Frankfurt will get cancelled. Airports in the Far East such as Korea and Bangkok experience no-relocation rates of 68.7% and 70.4%, respectively, mainly due to the low frequency of aircraft movements in the sample period. This results clearly illustrates the global impact of the event, which hints at the potential interest of airline alliances, such as Star Alliance, to also contribute to the investment to prevent disruptions across the alliance’s main global hubs.

Thus, the main conclusion is that, in view of the revenue loss for FRA, the acquisition of a counter-drone system can be justified when considering the possibility of several drone occurrences over time as current statistics show. Bringing airline costs into consideration, the decision becomes even more justifiable as Lufthansa could have a big interest in contributing to protect against high welfare costs and avoid customer dissatisfaction. Furthermore, it should be noted that real costs of a drone-related closure should be higher if we added arriving passengers and costs at other locations. From the current data analysed, the authors found that getting the support of airline alliances and increasing seat capacity after reopening are more effective than high-speed rail options in order to reduce passenger delays.

## Summary

This research aims to identify whether the investment cost of a counter-drone system at Frankfurt airport can be justified in comparison to the total cost experienced by the airport and airlines as a result of a complete shutdown due to a drone sighting in a period of peak activity similar to the 2018 Gatwick incident. Our method identifies the affected passengers for different closure scenarios and a custom-designed algorithm then searches for the next possible alternative connection with available seat capacity. Welfare costs for airlines consist of meal vouchers and hotel stays for the waiting time passengers spend on the terminal. Costs for the airport operator relate to loss of landing and passenger charges due to the cancellation of flights.

The findings revealed that the worst-case scenario of a continuous 48-h closure caused approximately €3.3 million in lost revenue for Frankfurt airport and €34.30 million in passenger welfare costs for the airlines. This only includes departing passengers at Frankfurt and thus, it can be considered a lower-bound estimate. Still, our results are sufficient to answer our main research question: the investment cost of a counter-drone system worth approximately €2.9 million per unit can be justified from the perspective of the airport operator (Fraport) based on the costs of a single 48-h incident during a peak period of activity. For two systems, the costs would be offset by the accumulation of shorter closure events for about 90 h. For further investments, dominant airlines may want to consider contributing funds since they are the ones bearing the highest costs. In the case of Frankfurt, Lufthansa alone experienced nearly 51.4% of the welfare expenses.

In relation to other airports, it is worth noting that Frankfurt is very well developed as an intermodal platform, which may explain why it has lower costs than Gatwick as it is easier for disrupted passengers to access alternative modes of transport. As Voltes-Dorta et al. (2017a) found, Frankfurt also is one of the most resilient airports in Europe and hence, the costs per passenger for other major European airports are likely to be even higher. Moreover, a larger presence of low-cost carriers in other hubs can make recovery worse due to the lack of alliance partners. As the number of consumer drone purchases is expected to increase in the future, it might only be a matter of time until the next major incident happens. On the other hand, with the strong increase in the number of counter-drone manufacturers on the market, there is also great potential for technology to develop and become more affordable. In addition, airports should not forget to implement other measures to complement anti-drone technology and possibly shorten the closures. For example, introducing a system to generate reports of drone activity from trusted sources, or engaging the airport’s closed-circuit video systems to identify the type of drone causing the disruption (Willis Tower Watson, 2019).

Still, the conclusions of our paper should be taken with caution due to several shortcomings. First, we ignore the cost for airlines relocating their cabin crew and pilots, providing accommodation and transport to other airports for cabin crew and pilots, repositioning aircraft that were diverted to other airports, or other long-term effects such as increased insurance premiums for airport and airlines due to more frequent drone attack occurrences. Third, future research could consider the knock-on effect to other airports caused by aircraft not departing from Frankfurt or passengers stranded at other airports waiting to depart to Frankfurt as local disruptions can have global impacts. At the same time, one may investigate the costs for service providers such as ground handling crews or the increased revenue for the airport in retail concessions due to longer passenger dwell times. Applying the methods developed in this research, one may rank airports worldwide according to their vulnerability to drone attacks in order to establish a typology of airports in which investment would be justified.

## Figures and Tables

**Fig. 5:**
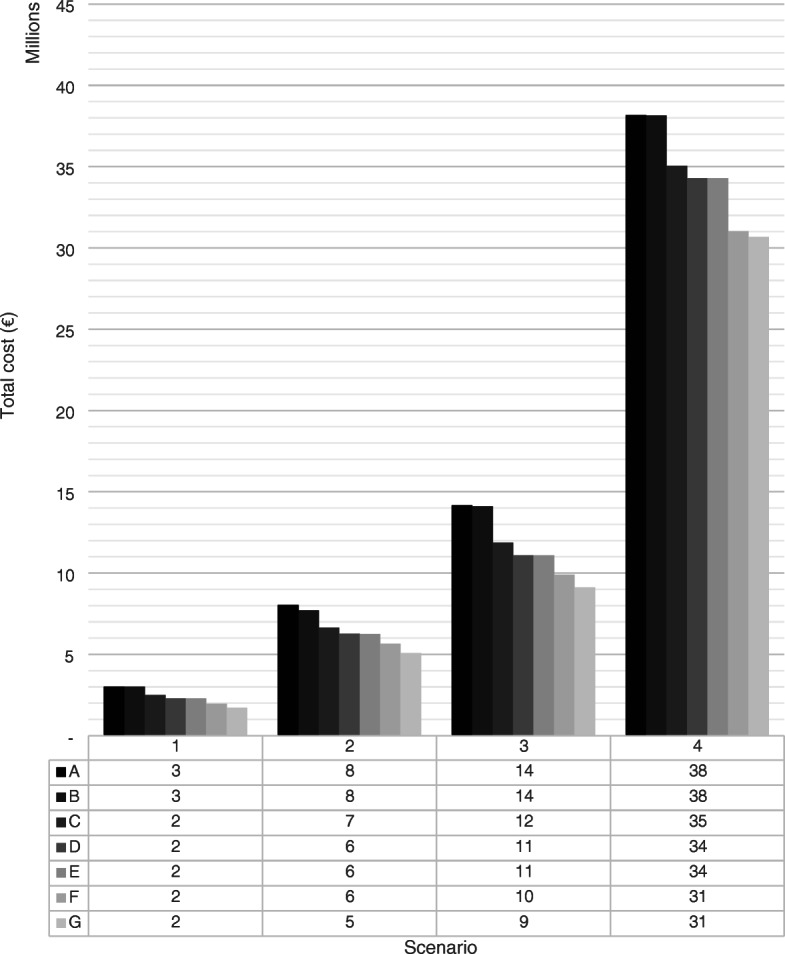
Total passenger welfare costs for all simulated closure scenarios and cases

**Fig. 6:**
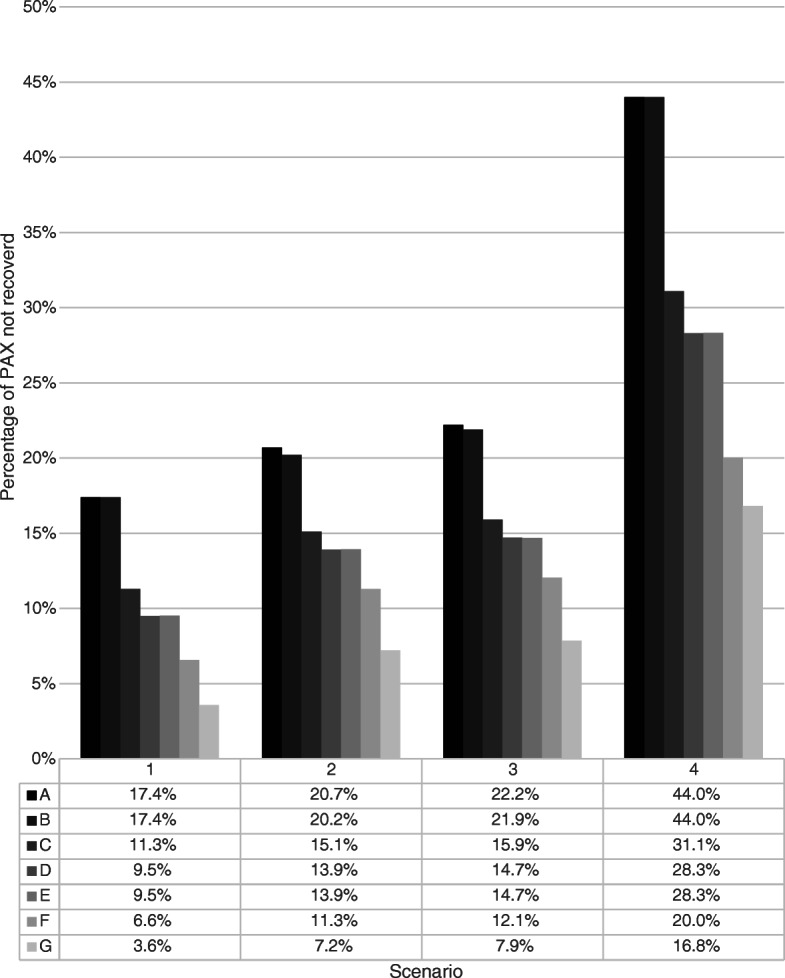
Proportion of non-relocated passengers for all simulated closure scenarios and cases

## Footnotes

1 Low-cost carriers (LCC) operate a limited variety of aircraft types. Hence, LCC flights will not increase capacity. Airlines serving long-haul international connections may be constrained in their capacity response as well. This will be covered by the sensitivity analysis.
